# Perityphlitis

**Published:** 1892-03

**Authors:** W. R. Blailock

**Affiliations:** McGregor, Tex.


For Daniel's Texas Medical Journal.

### PERITYPHLITIS.

W. R. BLAILOCK, M. D., M'GREGOR.

[Read at the Austin District Medical Society, December 24, 1891.]

THE RIGHT iliac fossa, and especially the vermiform appendix, instead of step-motherly treatment, as Kraussold complained, had been accorded it by the older anatomist, is receiving the attention usually given a rich and infirm spinster aunt.

Perhaps no subject pertaining to medicine or surgery has been more discussed during the past two years than diseases in and around the cæcum.

By common consent these diseases have been designated indiscriminately as typhlitis, appendicitis, perityphlitis, etc. This mixing of terms often leads, apparently, to wide differences of opinion, when really there is not much difference after all. The surgeon speaks of an appendicitis requiring a laparotomy. The physician or brother surgeon speaks of an appendicitis with his thoughts upon a typhlitis, condemns laparotomy, and cures his case by rest, purgation, restricted diet and opium.

The preponderance of opinion among surgeons of the widest experience in this field is that the great majority of diseases located here have their origin in the vermiform appendix. The importance, then, of correct anatomical knowledge of this organ and its relations to the cæcum and peritoneum cannot be overdrawn.

The vermiform appendix arises normally from the posterior inferior aspect of the cæcum, passes inward and upward behind the cæcum, is from three to six inches long, the size of a goose quill. Its canal is small and communicates with the cæcum by an orifice which is sometimes guarded by an incomplete valve.

Its coats are thick and its mucous lining furnished with a large number of solitary glands. (Gray.)

Sometimes it is abnormally long or short, open or closed, with a valve cylindrical saccular or bulbed, fixed or free, curved or bent upon itself at a sharp angle, provided with a short mesentery and sometimes entirely absent. (Kraussold.)

Dr. Randolph Winslow says: "It was supposed until quite recently that the cæcum was only partially covered by peritoneum leaving a large part of its wall with no peritoneal coat, and in relation with the post cecal connective tissue. This is an error; the cæcum has a distinct mesentery in the vast majority of cases and floats quite freely in the peritoneal cavity. When an abscess forms in the course of an appendicitis, pus is found primarily in the peritoneal cavity and not in the post cæcal connective tissue. It has been shown that the appendix is frequently the seat of ulcer or stricture somewhere in its course, the result of dysentery, typhoid fever, syphilis, and especially of tuberculosis. Normally the vermiform appendix is filled with mucus, but often masses of feces, foreign bodies, and intestinal worms find their way into it.

"Two anatomical factors have been cited to explain the frequent origin of disease in the appendix. One is the existence of the valvular fold of mucous membrane near the orifice of the tube in the cæcum. This fold is most marked between the ages of three and twelve, sometimes narrowing the orifice to one-half or one-thrid the calibre of the tube.

"The other is the deformity, caused by the abnormal anatomical position of the organ.

"Appendicitis occurs oftenest in young persons of the male sex.

"Constipation and sedentary habits have been invoked as causes of this disease, but when it is remembered that old persons and women are more prone to constipation and sedentary habits, and less prone to appendicitis and its associate lesions, they have to be given up as untenable.

"Perhaps tuberculosis stands in a greater causative relation to this disease than other agencies. The tubercle-bacilli, finding a suitable nidus for development in the numerous solitary glands of the appendix.

"This disease is usually ushered in with a chill followed by sharp pain in the abdomen, vomiting, thirst, fever, loss of strength, and an expression of great anxiety.

"A constant deep-boring lancinating pain, accompanied by induration in the right iliac fossa is regarded by many as the only symptom that can be relied upon.

"If the bowel move freely and the induration increase or remain stationary, and the fever continue, there is usually pus present.

"However, in peri and perityphlitis induration may not be discoverable by external palpation alone, but detected if conjoined with rectal examination.

"In typhlitis stercoralis the tumor in the flank soon becomes very pronounced, extending up the colon. The appendix is sometimes diseased without producing very marked symptoms. The McBurney point is universally condemned as being unreliable, owing to the varying position of the appendix.

"With very few exceptions present day surgeons condemn the hypodermic needle, for diagnostic purposes, around the cæcum. All information desirable from that source can be more safely obtained in other ways. It seems, however, the matter of diagnosis is comparatively easy, as no serious mistakes have been reported. Many cases have been opened with the expectation of finding other disorders, and the appendix found diseased. I do not wish to convey the idea that in all cases where appendicitis exists, it is easy to make a diagnosis, but where the symptoms are well marked, there is little danger of going wrong.

"In the treatment of these affections, when signs of sudden rupture of an abscess into the peritoneal cavity occurs, physicians and surgeons all agree, that, unless an operation is done, and perhaps even then, the undertaker will have work to do."

Mr. Trevis says, "Nearly all cases of typhlitis recover under ordinary medical treatment, and that only a few cases of appendicitis in young persons require operation. Probably no case should be operated upon before the fifth day, for cases of death on the third or fourth day are almost unknown."

Dr. Thos. S. K. Morton says: "Operate not later than the third day of the disease, if the patient up to that time has failed to improve under rest, restricted diet, purgatives, and topical application. Especially should this rule be adhered to where we fail to move the bowels. When the presence of pus is assured, where peritonitis is developing or spreading, etc."

Dr. Price says: "Operate fully for appendicitis, unless you can purge the bowels."

Dr. William Pepper says, "for more than a quarter of a century he has seen and treated numbers of cases of appendicitis each year, and believes, if every case were operated upon, the mortality would be tenfold what it is now. If signs of suppuration develop later on the simple Willard Parker extra peritoneal incision may be made.

In the light of the recent discovery of the relations of the cæcum to the peritoneal cavity, and the primary seat of pus in appendicitis being intra instead of extra peritoneal, Dr. Pepper's advice does not seem the best course to pursue.

Cases operated upon during the first forty-eight hours of the disease show a very small death rate. It is reasonable to assume that these cases receiving early operation may be classed with the most violent and dangerous cases. After forty-eight hours the death rate has increased rapidly.

Apropos of the Willard Parker extra peritoneal incision the following case is given:

For the early and recent history of this case, I am indebted to my friend Dr. J. E. Brown, of McGregor, who made a diagnosis of appendicitis that has since been verified, and advised an operation some time before it was done.

L. D. N., physician, age 24, previous health good, except at age of 12 had an abscess which was diagnosed hepatic abscess; it ruptured through the lung at some point on the back; discharged pus several months. History of tuberculosis in the family.

May 23, 1891, feeling of malaise; constipated for two days.

May 24, severe chill, accompanied by vomiting and griping pains in abdomen, followed by fever which rose to 103° F.

Hypodermic of morphia relieved pain to some extent, but tenderness on pressure existed in right iliac fossa. Tenderness increased, the bowels became tympanitic, and temperature reached 104° during the evening.

May 25, temperature 103°; increased tympanitis; some resistance in right flank. Epsom salts given, but not retained. Morphia repeated frequently; hot stupes applied. Evening, temp. 105; constant dull, boring, deep-seated pain in region of cæcum. A quantity of warm water in which 1 oz. Epsom salts was dissolved was thrown high up into the bowel. A copious evacuation of feces followed which, to a great extent, reduced the tympanitis and relieved the pain.

May 26, morning temp. 102; some pain and tenderness all over abdomen but more pronounced in right iliac fossa, where a well marked tumor could be made out. Another enema of warm water was given, producing a small fecal discharge. Evening, temperature 103; bowel tender and tympanitic. By end of second week temperature came down to normal. Tenderness disappeared by end of third week.

On attempting to walk, had pain in right hip, and felt as if the right side were too short. After a two weeks trip to the Gulf coast he resumed practice. One week afterward, pain in side returned; evening temperature above normal; loss of flesh and strength followed. Boring pain in right side; also pain in hip joint and down inside of thigh. Standing, he presented typical posture of hip joint disease.

These symptoms grew worse until July 12, 1891, when it was decided that an operation was imperative. Temperature 103. External palpitation did not reveal the presence of a tumor in right iliac fossa, but by introducing a finger into the rectum and placing the other hand over the cæcum a decided induration could be made out. This induration was aspirated but no pus found. Assisted by Drs. Brown and Young an incision about 3½ inches long parallel to Poupart's ligament, about 1½ inches in front of the anterior, superior spinous process of ilium down to the peritoneum was made. Dissected backward and outward with the finger until the posterior part of the cæcum was reached. A hard, tough feeling mass was here encountered attached to a part of the posterior cæcum. No pus was found.

A large drainage tube was passed down to the bottom of the opening, the wound closed up to the tube and dressed.

On the morning of the 14th the under part of the dressing was found saturated with pus. Pus continued to discharge freely; temperature came down to normal; pain disappeared entirely.

After about ten days, without any decrease of pus, fever and pain returned, and in spite of everything that was done, fever continued to recur, although a free discharge of pus went on all the while. In five or six weeks after an unusually free discharge of pus, his fever went higher, then came down to normal; pus ceased altogether. Patient could sit up; had a good appetite and there was every indication of a speedy recovery.

After a few days, fever returned; patient's condition no better than three weeks after the operation. The wound was washed out with hydrogen peroxide repeatedly, but no improvement. Thierch's solution was used, but aside from first washing, did no good. This state of affairs continued until Sept. 27, when another opening was made on the back, just above the crest of the ileum.

This point was tender and showed some bulging. When this opening was made there was no difficulty in introducing the finger into the bottom of the previous opening. Water thrown into the anterior opening flowed freely from the posterior one. A drainage tube was put in, and pus was soon flowing through it. This operation had no appreciable effect on the fever.

In November he went to New Orleans, and on the 12th Dr. Miles made three more openings,—one about two inches above, the other two inches below the opening on the back, the other midway between the anterior and posterior openings on crest of ileum—the latter for the purpose of scraping the crest of the ileum, which was found slightly necrosed, as was also the spine of one of the vertebræ. A concretion of fecal matter about the size of a hulled peanut was found which Dr. Miles said had ulcerated through the appendix and the opening closed by cicatrization.

Since the last operation his temperature has been as high as 103° but the last heard from him it was 99½. Dr. Miles did not think it necessary to remove the appendix.

I believe, instead of making the extra peritoneal incision, if I had opened up the peritoneum and removed the appendix, the result would have been better. No doubt if it had been done early in the first attack he would have been spared much suffering.

When surgeons learn to differentiate between an inflammation involving the cæcum, and one involving both cæcum and appendix, or the latter without the former, the indications for treatment will be clearer. During the first few days of the attack this seems impossible.

As the appendix is the starting point in most of these diseases, I think it would be well whenever practicable to remove it, even in operations for the removal of pus in peri and perityphlitis. Dr. Morton's rule to operate not later than the third day if no improvement results from purgation, topical appliances, rest and restricted diet, is worthy of careful consideration.

In securing purgation, an expedient that has given me good results, is to attach a flexible tube 18 to 36 inches long, and round pointed, to the nozzle of a syringe, place patient on right side, with hips elevated, introduce the tube its full length and distend the bowel with warm water, well impregnated with bland soap.

Sometimes it is necessary to repeat this a few times before the colon and cæcum are thoroughly emptied.

I have seen a number of ugly cases give way under this treatment. But in a number of cases which investigation proves to be relatively great, all efforts at medical treatment fail to bring about improvement. In these cases the rule above given applies.

